# The Inactivation of a New Peptidoglycan Hydrolase Pmp23 Leads to Abnormal Septum Formation in *Streptococcus pneumoniae* 

**DOI:** 10.2174/1874285800802010107

**Published:** 2008-08-22

**Authors:** Pagliero E, Dublet B, Frehel C, Dideberg O, Vernet T, Di Guilmi AM

**Affiliations:** 1 Laboratoire d'Ingénierie des Macromolécules; 2 Laboratoire de Spectrométrie de Masse des Protéines; 3 INSERM U570, Faculté de Médecine Necker-Enfants Malades, 156 rue de Vaugirard, 75730 Paris cedex 15, France; 1,2,4 Institut de Biologie Structurale Jean-Pierre Ebel (CEA-CNRS UMR 5075-UJF), 41 Rue Jules Horowitz 38027 Grenoble cedex 1, France

## Abstract

The bacterial peptidoglycan is the major component of the cell wall which integrity is essential to cell survival. In a previous work, we identified, in the positive-Gram pathogen *Streptococcus pneumoniae* , a unique protein containing a new putative peptidoglycan hydrolytic domain named PECACE (PEptidoglycan CArbohydrate Cleavage Enzyme). In this study, we characterise the physiological function of this protein called Pmp23 (Pneumococcal Membrane Protein of 23 kDa). A cell wall hydrolytic activity is observed with the recombinant protein. Inactivation of the *pmp23* gene in the pneumococcus led to a decreased flocculation, an increased sensitivity to β-lactam antibiotics and morphological alterations affecting the formation and localisation of the division septa. Taken together these observations indicate that Pmp23 is a hydrolase whose function is linked to peptidoglycan metabolism at the septum site.

## INTRODUCTION

The external bacteria cell wall gives the cellular morphological shape and allows resisting to the intracellular pressure. These properties are due to the main component of the cell wall, the peptidoglycan. This strong scaffolding structure is formed by glycan strands and peptide chains held together by covalent bonds, resulting in a mono- or multilayered network. The glycan strands are composed of *N*-acetylglucos-amine and *N*-acetylmuramyl residues linked together by β-1,4 glycosidic bonds. Peptides are covalently attached through amide bond to the lactyl group of the muramic acid and their cross-linking results in the net structure of the peptidoglycan.

The final steps of the peptidoglycan synthesis take place in the extracellular space and are catalyzed by the Penicillin-Binding Proteins (PBPs) anchored in the cytoplasmic membrane. The optimal peptidoglycan metabolism requires also the participation of hydrolases, which must function in concert with the PBPs. Even though their precise roles are poorly understood, these enzymes participate in fundamental biological functions by allowing the insertion of new material into the existing peptidoglycan and by triggering cell separation following division. The hydrolases contribute to peptidoglycan degradation and recycling, and participate in autolysis under certain growth conditions. They remodel the peptidoglycan to facilitate the passage of macromolecular transport systems [1].

*Streptococcus pneumoniae* is a pathogenic agent, which is responsible for over one million yearly deaths worldwide, causing pneumonia, otitis media, meningitis and sepsis cases, notably in the very young and in the elderly population. Infection by *S. pneumoniae* has been classically and successfully treated with β-lactam antibiotics, which inhibit the PBPs peptidoglycan synthetases. However, the increase in multi drug-resistant strains worldwide is a large medical and social problem as 25% of all invasive *S. pneumoniae* strains are today resistant to penicillins [2]. The future will most probably reside in the development of new drugs targeting bacterial essential processes.

The pattern of peptidoglycan hydrolases in *S. pneumoniae* includes a low molecular weight PBP with a D,D-carboxypeptidase activity involved in the regulation of the bacterial division and five cleaving enzymes. Four of these, LytA, LytB, LytC and CbpD are cell wall-associated proteins harbouring Choline-Binding Domains interacting with choline residues present on cell wall teichoic and lipoteichoic acids [3, 4]. LytA is an autolytic amidase causing bacteriolysis in stationary phase and in the presence of antibiotics [5]. LytB is a glucosaminidase involved in cell separation as a *lytB* mutant forms very long pneumococcal chains of over 100 cells [6, 7]. LytC is a lysozyme with an autolytic behaviour at 30°C [8]. Finally, CbpD and PcsB contain a CHAP domain (Cysteine, Histidine-dependent amidohydrolase/pep-tidase) predicted to cleave a peptidic bond but definitive biochemical data are still lacking [9,10]. These hydrolases play various roles such as allolysis in the competence process for LytA, LytC and CbpD [11, 12] and colonization of the nasopharynx, the initial step of the pneumococcal pathogenesis for LytB, LytC and CbpD [13]. While LytB is involved in cell separation during the late step of division, PcsB, the only essential pneumococcal hydrolase to date, appears to be involved in the early stages of the division since under-expression of PcsB leads to the formation of misplaced septa and large cells [14].

In a previous work, we have identified in the pneumococcus genome a new domain harbouring motifs that infer potential peptidoglycan cleavage activity. For this reason we named this domain PECACE (PEptidoglycan CArbohydrate Cleavage Enzyme). This domain was found exclusively in Gram-positive bacterial species; furthermore, its genetic organization is conserved among streptococcal species, suggesting a significant cellular role for the PECACE function. In addition, the PECACE domain is in various instances found in association with other domains known to catalyze peptidoglycan hydrolysis: this observation reinforces the predicted function of PECACE as participating in peptidoglycan cleavage and represents another example of multifunctional proteins involved in peptidoglycan metabolism. The PECACE domain identified in the pneumococcus genome is present in a sequence containing 204 amino acids (predicted molecular weight of 23 kDa), which harbour a short cytoplasmic region, a transmembrane anchor and a large extracellular region; this protein has been named Pmp23 for Pneumococcal Membrane Protein of 23 kDa.

We describe here the functional characterization of the Pmp23 protein in the* S. pneumoniae* R6 strain.

## MATERIALS AND METHODOLOGY

### Cloning of *pmp23* gene from the *S. pneumoniae * R6 strain

Genomic DNA from R6 strain was used as a template to amplify the complete *pmp23* gene containing the cytoplasmic region, the transmembrane anchor and the periplasmic domain. The upstream 5'-CGCGGATCCTTTAAACGAA TTCGAAGAGTGCTTGTACTAGCAGTC-3’ and downstream 5'-CCGCTCGAGCTAGCCAGATGTTGAAAAGA GAGTGAAACATTTGATGAT-3’ primers have respectively *BamHI *and *XhoI* sites (underlined). pGEX-4T-1/*pmp23* plasmid was obtained by cloning the PCR product into pGEX-4T-1 (Amersham Biosciences) and the absence of mutations into the *pmp23 *gene was checked by sequencing (Genome Express, Grenoble).

### Inactivation of *pmp23* gene in the *S. pneumoniae *R6 strain

Upstream and downstream flanking regions of *pmp23* (–504 to +307 and +399 to +1026 positions, respectively) were amplified by PCR and cloned into the FW13 plasmid; the cloning sites are on both sides of the *aacA/aphD* cassette encoding for kanamycin/gentamycin-resistance [15]. *S. pneu-moniae* R6 strain was transformed with 50 ng of pFW13/ *pmp23*. Colonies resulting from homologous recombination were selected on blood agar plates in the presence of 300 µg/ml of kanamycin. Inactivation of the *pmp23* gene was verified by PCR using external primers and PCR products obtained have been sequenced. The *pmp23::kan* R6 mutant strain was grown in Bacto^TM^ Todd Hewitt broth (TH broth) (BD Sciences) until an optical density at 620 nm (OD_620_) of 0.2 was reached. Cells were spun at 3,000 x *g* for 15 min and resuspended in TH broth containing 25% glycerol in order to concentrate the cellular suspension twice and 2 ml-aliquots were stored at -80°C.

### Deletion of *lytA* and *lytC* Genes in R6 and *pmp23::kan* R6 Strains

The phenotype of the pneumococcal R1547 strain is *lytA::cam, lytC::tet, pmp23::kan *[9]. The resistance cassettes (*lytA::cam* and *lytC::tet*) have been used for transformation of the R6 strain in order to generate single and double deletion mutants: *lytA, lytC, lytA/pmp23 *and *lytC/pmp23.* Antibiotic concentrations used for the selection of transformants were: chloramphenicol, 4.5 µg/ml; tetracycline, 0.5 µg/mL and kanamycin, 300 µg/ml. For each strain, the presence or the absence of *lytA*, *lytC *and *pmp23* genes was checked by PCR. Mutants were stored as previously described for the *pmp23::kan* R6 mutant strain.

### Growth Rate Measurements and Cell Viability Assay

2 mL cultures of *S. pneumoniae *with an OD_620_ of 0.4 were used to inoculate 50 ml of TH broth or glucose-buffered broth (Diagnostics Pasteur) at 37 °C. Whenever cell flocculation was observed, the OD_600_ reading was performed on the upper region of the unmixed culture tube. When required, 0.06 µg/ml of Penicillin G were added when the cultures reached an OD_620_ of 0.3-0.4. Assays of cell viability were performed by serial dilutions: 100 µl of cell suspension were deposited on Petri dish overlaid with 20 ml of Columbia blood agar base EH (BD Sciences) containing 4% horse blood (Eurobio) before incubation for 18h at 37°C.

### MIC Testing

The Minimal Inhibitory Concentration (MIC) for different β- lactams was measured three times using the E-test method (AB Biodisk). When different discrete values were measured, the results were expressed as a range of the maximal and minimal values.

### Morphological Characterization

Optical fluorescence microscopy was used to observed morphological changes in the various R6 mutant strains. Cells in exponential phase were fixed in 2.5% (v/v) paraformaldehyde, 0.03% glutharaldehyde, 30 mM sodium phosphate (pH 7.5) for successively 15 min at room temperature and 45 min on ice. Cells were washed three times in PBS pH 7.4 and resuspended in 50 mM glucose, 20 mM Tris-HCl pH 7.5, 10 mM EDTA. 30 µl of cell suspension were deposited on polylysine slides (Sigma), unbound cells were washed with PBS and a final fixation-permeabilization step was performed using ice-cold methanol for 5 min. A solution of fluorescein isothiocyanate (FITC) (Sigma) at 200 µg/ml was deposited on the fixed cells and was incubated for 45 min at room temperature. Labelled cells were washed 5 times with PBS and mounted in Moviol. The observations were performed with a 100 X immersion objective on an Axioplan 2 fluorescence microscope (Zeiss) equipped with axiocam MR imaging system and axiovision software (Zeiss).

Transmission electron microscopy was performed on ultra-thin sections of wild-type and *pmp23::kan* mutant R6 strains using with a JEOL 100-CXII electron microscope. Cell samples cultured in glucose-buffered broth at 30°C were taken from early and late exponential phases and processed as previously described [16]. Briefly, after centrifugation at 7,000 x *g* during 3 min, the bacteria pellet was fixed overnight at 4°C in a solution of 2.5% glutaraldehyde in TEM buffer (100 mM sodium cacodylate pH 7.2, 5 mM CaCl_2_ and 5 mM MgCl_2_). Fixed-cells were washed twice in TEM buffer, post-fixed for 1h in 1% osmium tetroxide in TEM buffer followed by an 1h00 incubation with 1% uranyl acetate dissolved in 100 mM veronal pH 6.8. The dehydratation was performed using acetone and the cell pellets were embedded in Epon. Thin sections were double-stained with uranyl acetate and lead citrate.

### Expression and Purification of Recombinant GST-Pmp23 protein in *Escherichia coli*

An overnight culture of an *E. coli* MC1061 expression strain transformed with pGEX-4T-1/*pmp23* plasmid was used to inoculate (1:50) 1l of rich medium for autoinduction (1 mM MgSO_4_, 100 mM (NH_4_)_2_SO_4_, 50 mM KH_2_PO_4_, 50 mM Na_2_HPO_4_, 0.5% (w/v) glycerol, 0.05% (w/v) glucose, 0.2% (w/v) b-lactose, 0.5% (w/v) yeast extract, 1% (w/v) tryptone, pH 6.8) supplemented with 100 µg/ml of ampicillin, the growth is performed at 30°C. The cells were centrifuged at 6,000 x *g* for 20 min when the culture reached an OD_600_ value around 10. All purification steps were performed at 4°C. Cell lysis was made by sonication in 50 mM Na acetate (pH 5.3), 200 mM NaCl, 10 mM EDTA, with a protease inhibitor cocktail tablet (Complete, Roche). The supernatant fraction obtained after low-speed centrifugation (2,000 x *g*, 15 min) was ultra-centrifuged at 184,000 x *g* for 1h00. The membrane-enriched fraction recovered in the pellet was resuspended in 50 mM Na acetate pH 5.3, 200 mM NaCl, 10 mM EDTA, and 2% Triton X-100 and stirred overnight before a second identical ultra-centrifugation step. The resulting supernatant, containing the detergent-solubilized GST-Pmp23 protein, was loaded at a flow rate of 1 ml/min onto a glutathione Sepharose column (Amersham Biosciences) equilibrated in 50 mM Na acetate pH 5.3, 200 mM NaCl, 10 mM MgCl_2_, and 0.5% Triton X-100. GST-Pmp23 was eluted with 10 mM reduced glutathione (Sigma) and dialysed against column equilibration buffer.

### Detection of Murein-Degrading Activity by the Zymogram Technique

Purified GST-Pmp23 protein was loaded on a 12.5% SDS polyacrylamide gel containing 0.023% (w/v) of *Micrococcus lysodeikticus* lyophilized cells (Sigma): the run was performed under denaturing conditions [17-20]. The gel was washed twice in distilled water for 30 min at room temperature, the in-gel renaturation of GST-Pmp23 protein was subsequently performed by incubating the gel for 48 h in 50 mM Na acetate pH 5.3, 10 mM MgSO_4_ 1% Triton X-100 at 30°C. The gel was stained with 0.1% methylene blue in 0.01% KOH and destained in water.

## RESULTS

### Predicted Topology of Pmp23

We have previously reported the identification of the PECACE domain putatively involved in peptidoglycan hydrolysis [21]. The probable enzymatic activity deduced from the detailed analysis of the amino-acid sequence suggested that the PECACE domain might proceed through a lytic transglycosylase-type with Slt70 as the paradigm enzyme or goose lysozyme-type cleavage mechanism. PECACE domain was exclusively found in Gram-positive bacteria alone or in association with others domains known to hydrolyse the peptidoglycan.

In *S. pneumoniae*, this domain is contained in a 204 amino-acid protein (calculated mass of 23 kDa), called Pmp23. The predicted topology is presented in Fig. (**1**): a short cytoplasmic peptide (M_1_ to R_7_) precedes the transmembrane anchor (V_8_ to G_18_) and the extracellular region (Y_19_ to G_204_) contains the PECACE domain (P_36_ to A_140_). Considering the membrane topology of Pmp23 and the putative enzymatic activity deduced from the amino-acid sequence, it appears that the Pmp23 function may differ from the other hydrolases already identified in *S. pneumoniae*, LytA, LytB, LytC, CbpD and PcsB. In order to shed light on the physiological function of Pmp23, the corresponding gene has been deleted by double homologous recombination in the R6 strain and the following properties have been analyzed to define the mutant strain phenotype: growth rate, β-lactam sensitivity and morphology.

### Growth Properties of R6 and *pmp23::kan* Mutant Strains

We compared the growth of the R6 and *pmp23::kan* mutant strains in TH and in glucose-buffered broths: the growth rate, 41 ± 2.5 min, and the autolysis profile were identical for both strains in either media culture (data not shown).

**Fig. (2).Flocculation measurements of the R6 parental and pmp23::kan mutant strains.**
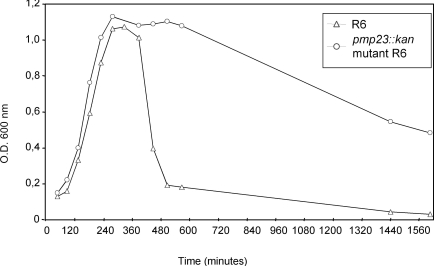
*S. pneumoniae* R6 strain (open triangle) and *pmp23::kan* mutant (open circle) were grown in glucose-buffered broth. One representative of at least three experiments is shown.

We then analysed the flocculation behaviour in glucose-buffered broth of R6 *S. pneumoniae *strain, which corresponds to the spontaneous cell aggregation and formation of a bacterial pellet leading to a decrease of the OD_600_ in the upper section of the culture tube. In these culture conditions, the R6 parental strain shows a rapid decrease of the OD_600_ value 100 min after reaching the stationary phase (Fig. **2**). On the contrary, the OD_600_ value of the *pmp23::kan* mutant strain remains at 1.1 for 3h after the beginning of the stationary phase and then, the OD_600_ decreases to a value of about 0.5 after 27h (Fig. **2**). It may be supposed that the specific flocculation behaviour of the *pmp23::kan* mutant strain is likely to be due to a modification of the cell wall main component, the peptidoglycan, as it is in direct contact with the culture medium in the absence of a capsule.

### Inactivation of *pmp23* gene increases β-lactam sensitivity

The antibiotic sensitivity of the R6 and the *pmp23::kan* mutant strains was analyzed by measuring the MIC values for various β-lactams using the E-Test method. The MIC values for cefotaxime or penicillin G are 0.025-0.038 µg/ml and 0.016 µg/ml for the wild-type and the* pmp23::kan* mutant strains, respectively. The MIC values for piperacillin are 0.032 µg/ml and 0.016-0.023 µg/ml for the R6 and the *pmp23::kan* mutant strains, respectively.

**Fig. (3).Effect of pmp23::kan mutation on penicillin-induced lysis and death.**
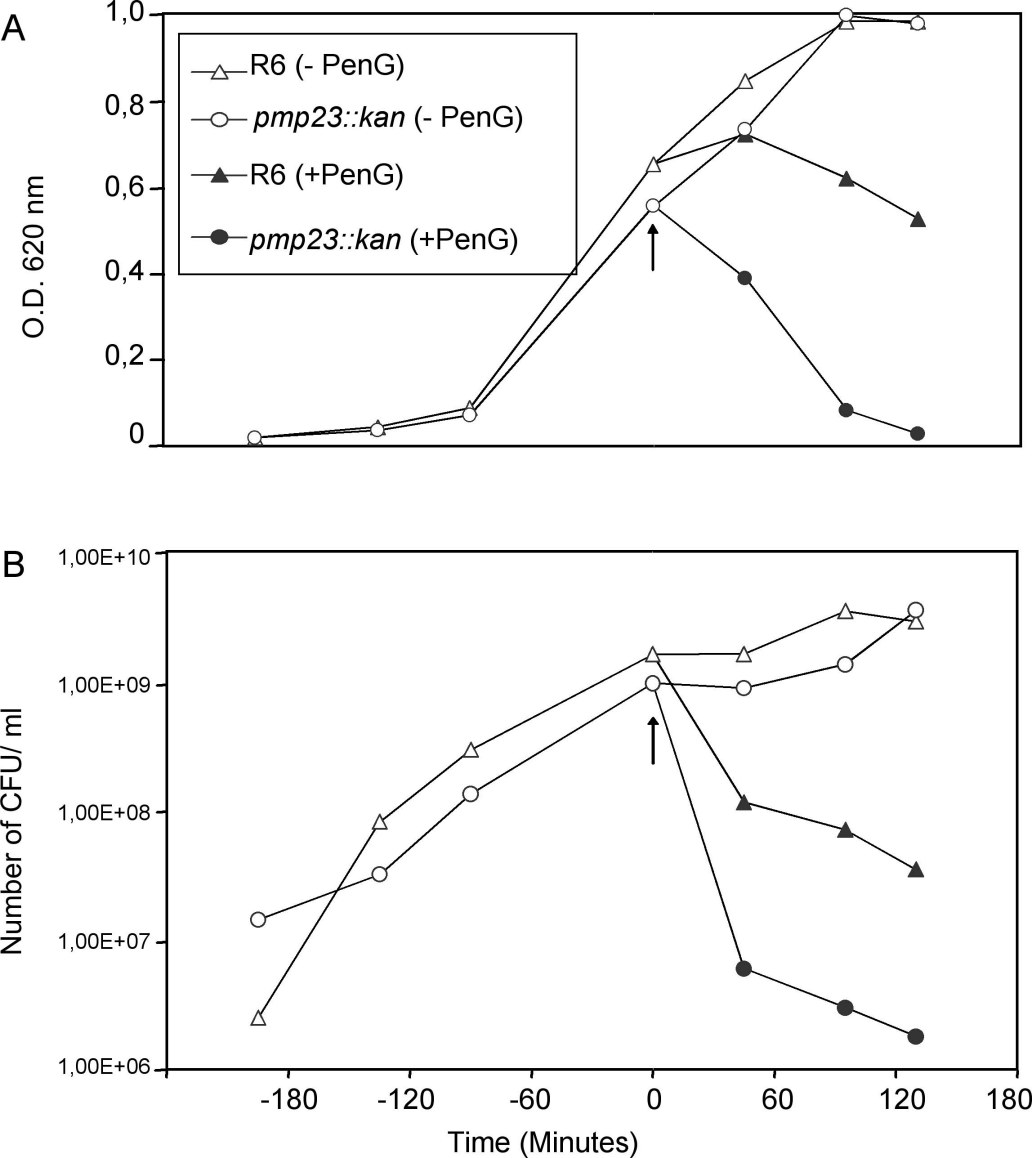
*S. pneumoniae* R6 strain (triangle) and *pmp23::kan* mutant (circle) were exposed (closed symbols) or not (open symbols) to 0.06 µg/ml of Penicillin G in TH broth. The arrow indicates Penicillin G addition. In each case, one representative of four experiments at least is shown. **A.** Lysis of *S. pneumoniae* R6 strain and *pmp23::kan* mutant in presence or absence of Penicillin G. **B.** Viability of *S. pneumoniae* R6 strain and *pmp23::kan* mutant in presence or absence of Penicillin G.

According to these results, the sensitivity towards penicillin G was then analyzed in liquid cultures: cell lysis (OD_620_ values) and the viability (colony forming units (CFUs) numbering on plates) values were measured in the presence of penicillin G (Fig. **3**). Both parental and mutant strains were grown in TH broth at 37°C, penicillin G was added at a concentration of 0.06 µg/ml when mid-exponential growth phase was reached and the culture growth was pursued for 2h post-penicillin G addition. In absence of penicillin G, the growth curves are identical for both strains, the stationary phases are reached concomitantly (Fig. **3A**, open symbols), and the cell viability for both strains is also comparable (Fig. **3B**, open symbols). The addition of penicillin G in the pneumococcal cultures induces a more rapid and pronounced lysis of the mutant strain than of the R6 parental strain (Fig. **3A**, closed symbols). Indeed, 130 min post-penicillin G addition, 97 % of the *pmp23::kan* mutant cells were lysed compared to the lysis of only 46 % of the parental cells. The pattern of cell lysis is correlated to the cell viability: in presence of penicillin G, the mutant strain is killed more rapidly and to a larger extend (one log difference) when compared with the parental strain (Fig. **3B**, closed symbols). In conclusion, the absence of a functional *pmp23* gene in *S. pneumoniae* increases β-lactam sensitivity.

### Altered Division Sites in the *pmp23::kan* Mutant Strain

**Fig. (4).Electron microscopy of thin sections of R6 parental and pmp23::kan mutant strains.**
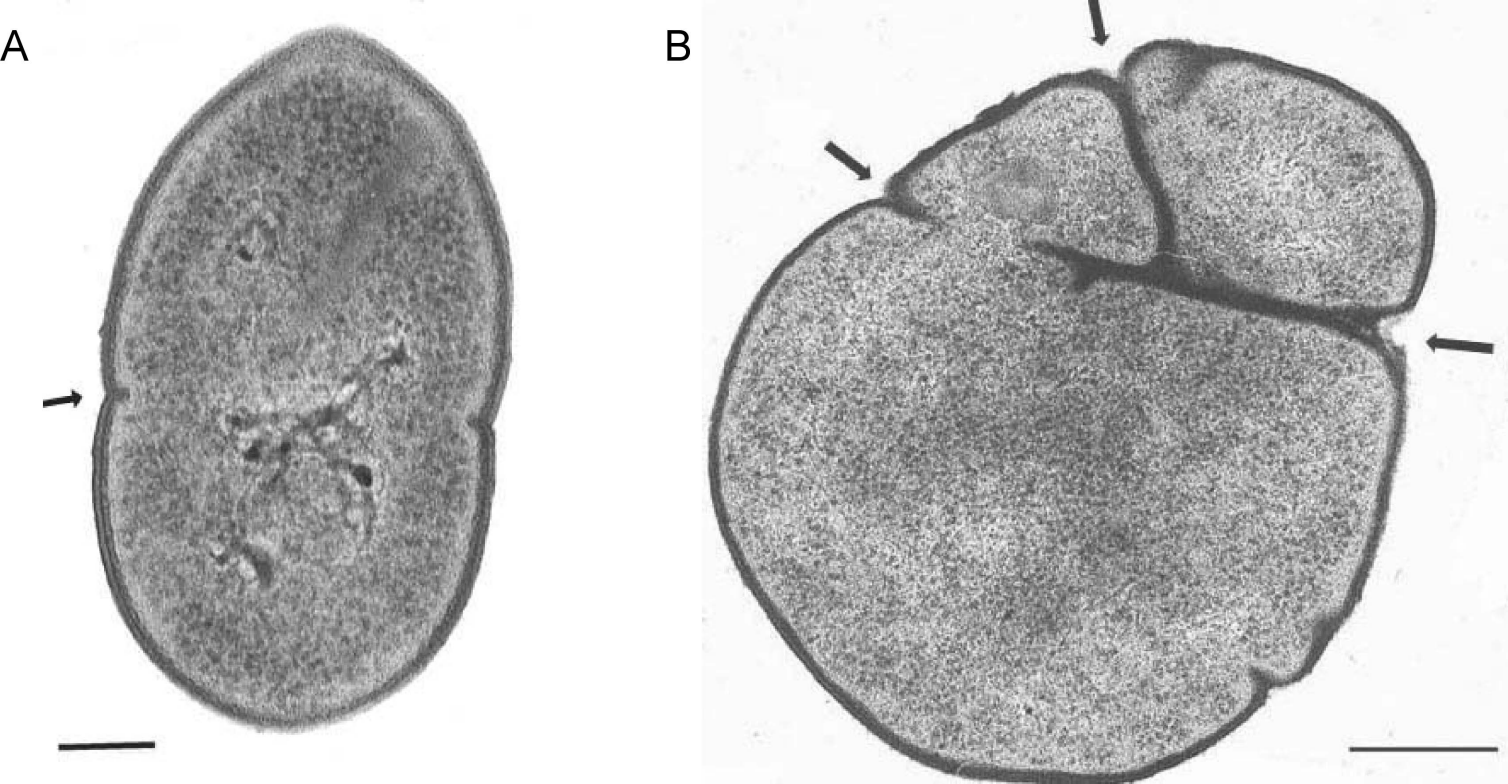
**A.** R6 strain bacteria displays the typical streptococcal morphology with regular shape due to symmetrical septum formation (arrow). **B.** *pmp23::kan* mutant harbors numerous and misplaced septa (arrows). Scale bar: 0,15µm.

Ultra thin sections of both R6 and *pmp23::kan* mutant cells were observed by transmission electron microscopy. As shown in Fig. (**4**), the *pmp23::kan* mutant strain displays morphological aberration when compared to the parental strain. The wild-type strain presents a typical streptococcal morphology with a regular shape and a symmetrical septum formation giving rise to two identical daughter cells (Fig. **4A**). The mutated strain shows abnormal shape due to a non-symmetrical septum formation or to the presence of several septa (Fig. **4B**). Observation by transmission electron microscopy of mutant cells negatively stained indicated that abnormal cells represent 15% to 20% of the total mutated population. This result indicates that Pmp23 may play a role in the positioning of the division site and/or maturation of the septum.

### Expression and Purification of a Recombinant form of Pmp23 Protein

**Fig. (5). GST-Pmp23 purification and zymogram.**
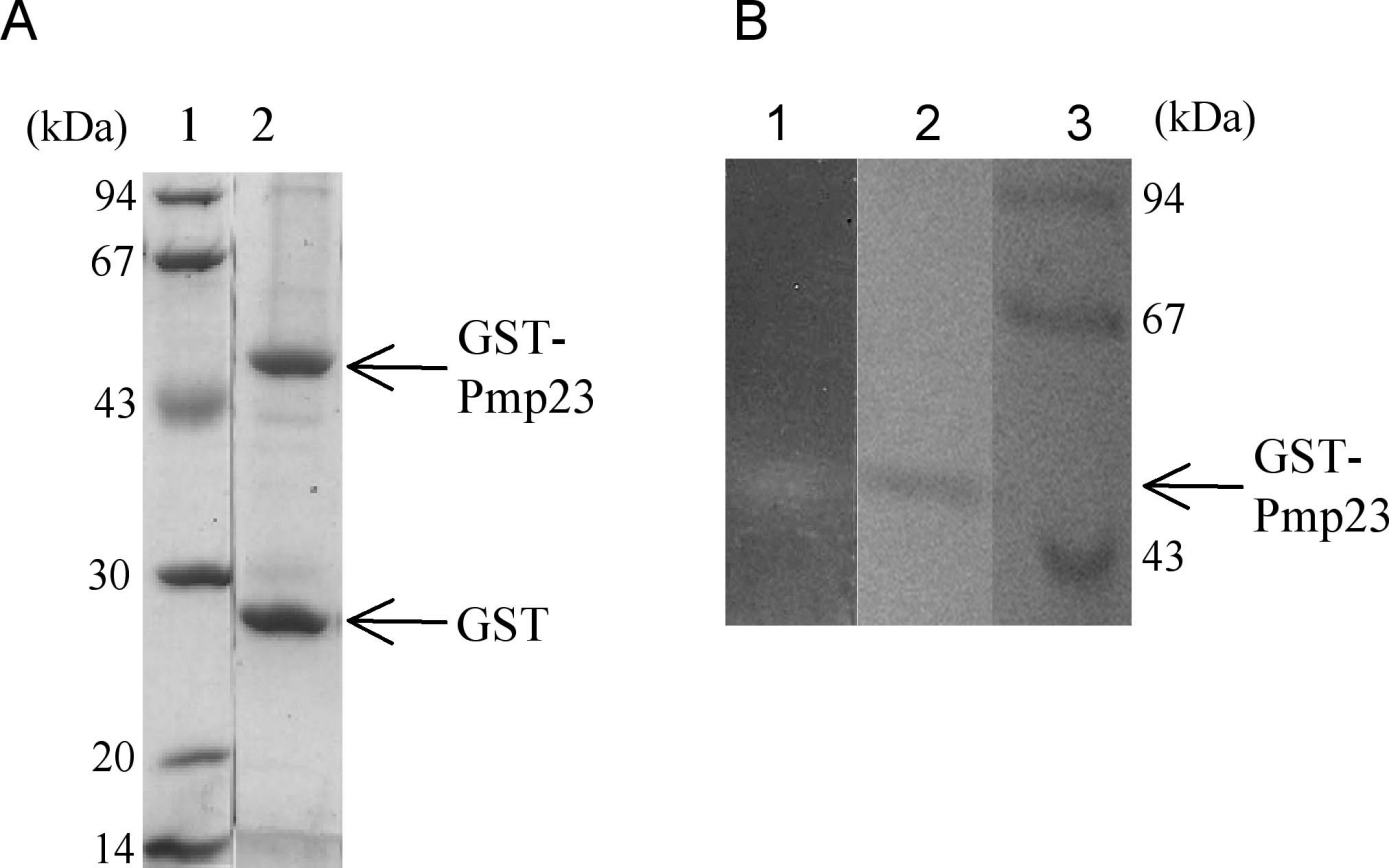
**A.** SDS-12.5% PAGE gel stained with Coomassie blue. Lane 1, standard molecular mass markers; lane 2, purified GST-Pmp23. **B.** Zymogram analysis: SDS-12.5% PAGE gel containing *Micro-coccus lysodeikticus* cells. Lane 1, renaturation of purified GST-Pmp23; lane 2, purified GST-Pmp23 stained with Coomassie blue; and lane 3, standard molecular mass markers

Pmp23 protein was expressed in *E. coli* in order to test its putative peptidoglycan hydrolysis activity. The protein was particularly difficult to express in a soluble form. Attempts at expressing the whole Pmp23 protein (residues 1 to 204) or the extracellular region (residues 36 to 204) fused to polyhistidin tags or to the signal peptide *pelB* (allowing the secretion into the periplasmic space of *E. coli*) delivered inclusion bodies. Low amounts of soluble protein was obtained when the extracellular region of Pmp23 was expressed fused C-terminal to the GST. The fusion protein co-purified with the GroEL chaperone, indicating that it was not properly folded. The expression of the full-sized protein linked to GST led to a fused protein that co-purified with the *E. coli *membrane fraction showing that Pmp23 is, as predicted, a membrane protein (data not shown). In order to increase the recovery yield, the GST-Pmp23 protein was extracted with 2% Triton X-100 from whole cell lysate. Affinity purification on a glutathione column was performed in the presence of 0.5% Triton X-100 [20, 22-24]. The apparent molecular weight of the purified GST-Pmp23 protein was 49.3 kDa in accordance with the calculated mass of 49.2 kDa (Fig. **5A**). As expected, this protein reacts against anti-GST and anti-Pmp23 antibodies (data not shown). The fusion protein is highly unstable and some 26.3 kDa GST moiety is always found free from the fusion (Fig. **5A**). All together, about 1 mg of GST-Pmp23 protein was purified from 1 litre of *E. coli *culture, a good yield considering the membranous property of Pmp23.

### Activity of recombinant Pmp23 protein

The cell wall lysis activity of Pmp23 was tested using the in-gel zymogram technique. Purified GST-Pmp23 was run on a SDS-polyacrylamide gel containing *Micrococcus lysodeikticus* lyophilized cells. Following protein renaturation, a clear band within the opaque background appeared at the location of the GST-Pmp23 protein (Fig. **5B1**). The contrast between the band and the background was enhanced by staining the gel with methylene blue (Fig. **5B2**). This experiment shows that the full-size Pmp23 protein fused to GST is functional as it has the ability to degrade a bacterial extract containing peptidoglycan.

### Phenotype analysis of the *pmp23* inactivation in *lytA *and *lytC* R6 mutant strains

In order to investigate the relationship between Pmp23 and the well-known pneumococcal autolysins LytA and LytC, the *pmp23* gene has been inactivated in R6 strains in which the genes encoding either autolysin have been inactivated. The growth rates have been measured for the single and double mutant strains compared to the parental R6 strain. Interestingly, the double *lytA/pmp23* and *lytC/pmp23* mutant strains display a growth rate of 40.5 ± 3.5 min similar to the parental strain while the single *lytA* and *lytC* mutant strains present a longer generation time of 47 ± 1.4 min (data not shown).

**Fig. (6).pmp23::kan mutation in lytA and lytC mutant R6 strains.**
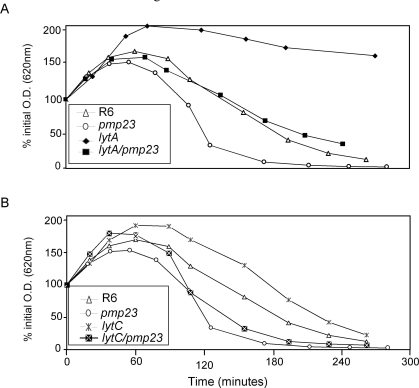
The strains were exposed to 0.06 µg/ml of Penicillin G in TH broth. The lysis of each strain was followed by monitoring the optical density at 620 nm. To compare the effect of Penicillin G on the different strains, the relative Penicillin G sensibility is presented as the percent of the initial OD_620_ measured just before penicillin addition. This experiment is representative of four assays. **A.** lytA background **B.** lytC background

The sensitivity to lysis induced by penicillin G has also been studied for all mutant strains (Fig. **6**). As already reported, the *lytA* mutant strain displays a reduced sensitivity to penicillin-induced lysis when compared to R6 strain [25-30] (Fig. **6A**). As already described in this work, the *pmp23::kan* mutant is more sensitive to penicillin G than the parental R6 strain. The double *lytA/pmp23* mutant displays an intermediate sensitivity between those of *lytA* and *pmp23* mutant strains (Fig. **6A**). The *lytC* single mutant is slightly less sensitive to penicillin G-induced lysis than the parental strain while the double *lytC*/*pmp23* strain harbours a lysis pattern more comparable to the *pmp23::kan* mutant strain (Fig. **6B**). These experiments have also been performed at 30°C and similar results have been observed (data not shown). These experiments suggest that the role of Pmp23 is independent from the functions of LytA and LytC, which are involved in cells separation at the end of the division process.

Cell separation was monitored by optical fluorescent microscopy following labelling of the cells. R6 and *pmp23::kan* mutant strains present 98% of single- and doubled-cell chains while this value is 75% for *lytC* and *lytC/pmp23* strains and 63% for *lytA* and *lytA/pmp23* mutant strains. The single and double *lytA* and *lytC* mutant strains display also 10 to 20% of chains containing from 4 to 20 cells. The triple mutant strain *lytA/lytC/pmp23* has been analysed: only 34% of the chains are singled or doubled cells, while a significant proportion of chains contains more than 50 cells. These data suggest that Pmp23 is not involved in the separation of the daughter cells after the achievement of the cellular division, as already mentioned above and tend to confirm that the physiological role of Pmp23 is distinct from the function of LytA and LytC.

## DISCUSSION

In a previous work, we have identified the membrane protein Pmp23 of *S. pneumoniae* that carries the PECACE domain [21]. The three-dimensional fold prediction of this domain is analogous to the catalytic sites of the lytic transglycosylase Slt70 from *E. coli* and of the goose-type lysozyme. This domain found exclusively in Gram-positive bacteria and located within conserved gene clusters in Streptococcal species, is sometimes found in association with other domains known to hydrolyse peptidoglycan like the CHAP the Nlpc/P60 and the peptidase M37 domains. The 204 amino-acid protein Pmp23 differs from the other pneumococcal hydrolases by its membrane topology: LytA, LytB, LytC and CbpD, all bind to the cell wall choline residues. As a transmembrane protein, Pmp23 is located near the site of peptidoglycan synthesis. The cell wall hydrolytic activity of Pmp23 is supported by our observation that purified GST-Pmp23 fused protein degrades *micrococcus* *lysodeikticus* extracts as observed on a zymogram.

The *pmp23* gene is not essential. The growth rate of *S. pneumoniae* deleted of the* pmp23* gene is similar to the parent strain and the lysis phase was unaltered showing that Pmp23 is not an autolysin. Indeed, analysis of single or combined *pmp23*, *lytA* and *lytC* mutants revealed that Pmp23 acts on a process that differs from those driven by autolysins LytA and LytC. We observe the following phenotypes associates with the *pmp23::kan* mutant strain: (1), a decreased flocculation; (2), an increased sensitivity to β-lactam antibiotics; (3), morphological alterations affecting the formation and localisation of the division septa. Taken together these observations indicate that Pmp23 is involved in defining the cell surface properties and peptidoglycan metabolism. Pmp23 is unlikely to be involved in major chemical modifications of the peptidoglycan. Indeed, the composition of the peptidoglycan of the R6 and *pmp23::kan* mutant strains is similar when analysed using a combination of RP-HPLC and mass spectrometry (data not shown).

The β-lactam sensitivity increase in the mutant strain may be indicative of a weaker peptidoglycan structure, even tough this does not correlate with a detectable chemical modification as mentioned above. One possibility would rely on the PBPs function: in absence of the Pmp23 enzyme, their physiological activity dedicated to peptidoglycan synthesis, could be altered leading to an immature and fragile peptidoglycan, more prone to induce bacterial lysis in presence of β -lactams. This putative coordinated function of Pmp23 with the PBPs may take place at the division site.

The absence of *pmp23* gene has a striking effect on position and aspect of the septum in a fraction of fast growing cells. This observation might be related to a specific degradation of the peptidoglycan by Pmp23 at inappropriate septum sites. An alternative, to this “quality control” hypothesis, would call for role of Pmp23 in defining the localisation of the septum and/or regulating the formation of the septum through peptidoglycan remodelling. This implies that Pmp23 interacts with some cell division proteins and/or septal PBPs. Similar interactions have been reported, in *E. coli* from which lytic transglycosylases and PBPs have been co-purified [31, 32]. Aberrant septum localisation in *S. pneumoniae *cells have been described previously for PBP1b/PBP2a and PBP1a/PBP1b double mutants [33]. The D,D-carboxy-peptidase PBP3 has also been shown to regulate the division site since the ∆*dacA* mutant displays multiple septa leading to cell widening and spherical morphology [34, 35].

When the essential peptidoglycan hydrolase PcsB is severely under-expressed, pneumococcus present abnormal cell wall synthesis at misplaced septa [14], a phenotype reminiscent to the one found in our *pmp23::kan* mutant. PcsB, contains a CHAP domain for hydrolysis of the peptide moiety of peptidoglycan whereas Pmp23 is probably involved in the cleavage of the glycan part. In fact, PECACE domains are sometimes found in association with CHAP domains [21]. It is tempting to propose that Pmp23 and PcsB act in coordinated way to regulate cell wall synthesis.

## CONCLUSION

Thus, Pmp23 is involved in peptidoglycan metabolism at the septum site. Further experiments are needed to establish the relationship between Pmp23 and other peptidoglycan hydrolases linked to cell synthesis and division.

## Figures and Tables

**Fig. (1).Schematic representation of the topology of Pmp23.**
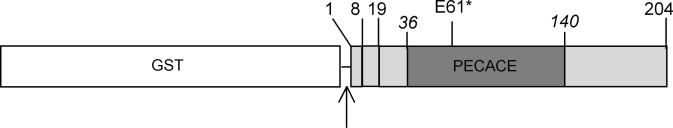
White box corresponds to the fusion protein glutathione-S-transferase (GST) and the thrombin site is indicated by an arrow. The light grey box figures the Pmp23 protein (residues 1 to 204) composed by: the cytoplasmic region (residues 1 to 7), the transmembrane anchor (resi-dues 8 to 18) and the PECACE domain (dark grey, residues 36 to 140). The asterisk indicates the potential catalytic glutamate E61.
